# Diagnostic imaging of deep vein thrombosis secondary to osteochondroma formation

**DOI:** 10.1259/bjrcr.20170042

**Published:** 2017-08-29

**Authors:** Brian M Moloney, Peter F McAnena, Donald G Courtney, AnnaMarie O’ Connell, William Curtin, Peter A McCarthy

**Affiliations:** ^1^Discipline of Surgery, Lambe Institute for Translational Research, National University of Ireland, Galway, Ireland; ^2^Department of Radiology, Galway University Hospital, Saolta University Healthcare Group, Galway, Ireland; ^3^Department of Trauma and Orthopaedic Surgery, Galway University Hospital, Saolta University Healthcare Group, Galway, Ireland

## Abstract

Deep vein thrombosis (DVT) is a condition classically associated with blood stasis, hypercoagulability or injury to the vasculature. As blood stasis is usually associated with patient immobility, DVT occurrence in young active patients with no underlying haematological conditions is a rarity. An exostosis, also known as osteochondroma, is a cartilage capped lesion. If solitary, they represent low malignant potential and unless symptomatic, they are rarely excised. A 23-year-old, active male, presented to hospital with pain and swelling in the left lower leg. It was a deep, non-radiating pain, exacerbated by exercise. Wells' criteria score for DVT was 2. An ultrasound was performed which identified thrombosis in the superficial femoral, and popliteal veins. Haematological causes of thrombosis were ruled out. X-ray showed a posterior femoral exostosis. It was determined that compression by the exostosis was the cause of the thrombosis. We present a case of a DVT secondary to osteochondroma formation in a young male. Isolated DVT in this setting is uncommon with fewer than five previously reported cases identified in the literature. We also discuss the current literature and management of this rare entity.

## Introduction

Deep Vein Thrombosis (DVT) is a common condition which occurs due to the presence of thrombus in a vein. Due to obstruction of venous pathways, patients frequently complain of symptoms such as pain, redness, warmth and swelling. DVT is therapeutically managed in a hospital setting due to the risk of pulmonary embolism, which is a potentially life-threatening condition.

Historically, three broad categories of factors are thought to contribute to thrombosis: venous stasis, hypercoagulability and endothelial damage. These factors are known as Virchow’s triad.

## Clinical presentation

A 23-year-old male presented to the emergency department complaining of pain and swelling of the left lower leg. He described a constant deep pain that was exacerbated by exercise that had been intermittently troubling him for over 1 month. On this presentation, however, the pain and swelling had occurred acutely over 12 h and persisted despite terminating exercise.

On examination, the leg was noted to be inflamed and the patient described tenderness throughout the lower limb. There was associated swelling, which was non-pitting in nature. The limb was significantly (4 cm) larger in size than the contralateral leg. With a Well’s score of 2, coupled with no history of immobility, it appeared highly unlikely that the young patient was suffering from deep venous thrombosis.

## Differential diagnoses

- Deep vein thrombosis
  - Venous stasis
    - Osteochondroma
    - Bakers cyst
    - Venous insufficiency
  - Hypercoagulability
    - Cancer
    - Antithrombin deficiency
    - Protein C deficiency
    - Protein S deficiency
    - Factor V Leiden
  - Endothelial damage
    - Smoking
- Venous Insufficiency

## Investigation/imaging findings

An ultrasound of the left leg was performed to investigate for deep vein thrombosis. Both the distal superficial femoral vein and the popliteal vein were noted to be occluded. The patient was subsequently treated with the therapeutic dose of low molecular weight heparin (14,000 IU Tinzaparin) and thromboembolic deterrent stockings, and the patient’s care was managed further by the haematological service. A thorough thrombophilia screen was performed. There was no haematological abnormality identified that could account for a deep vein thrombosis.

**Figure 1.**
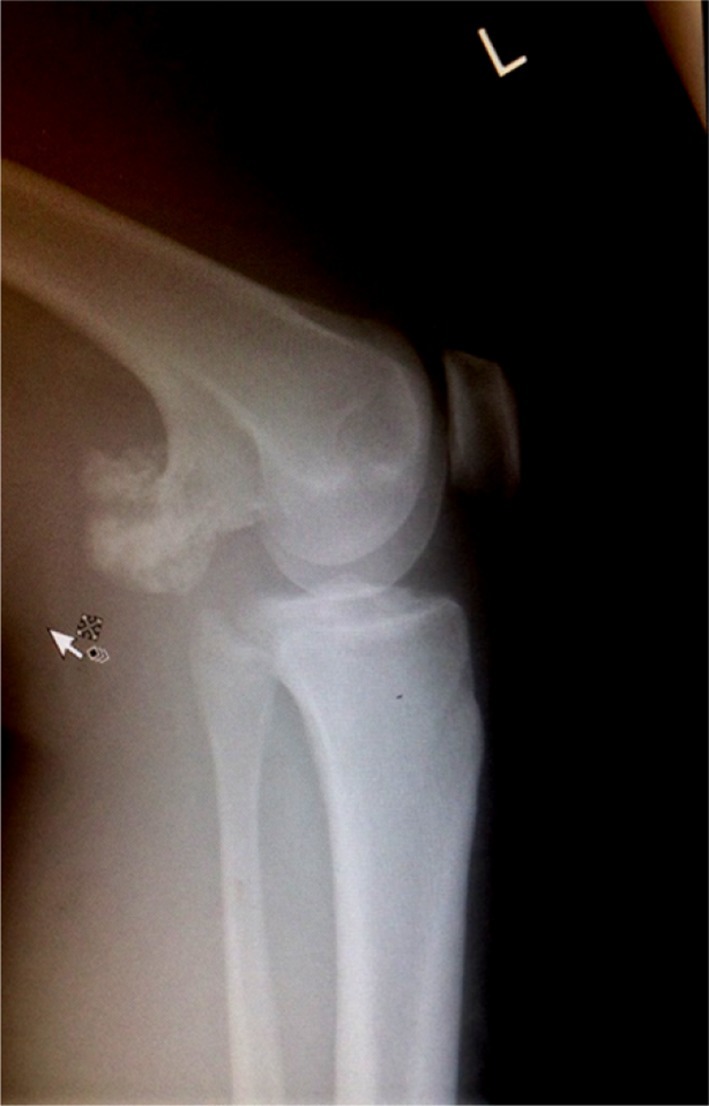
X-ray imaging revealed a large bony growth originating from the distal femur in the popliteal fossa.

With no improvement of clinical findings following prolonged therapeutic thrombolytic treatment, a repeat of the Doppler ultrasound was performed. Ultrasonography revealed that the thrombosed veins had not recanalized. A plain film radiograph of the knee, however, indicated that the patient had a small femoral bony exostosis in the popliteal fossa (Figure 1). A multidisciplinary approach was employed, involving orthopaedic, vascular and haematological services. It was determined that an excision of the exostosis of the distal femur would produce the best outcome.

## Treatment

**Figure 2.**
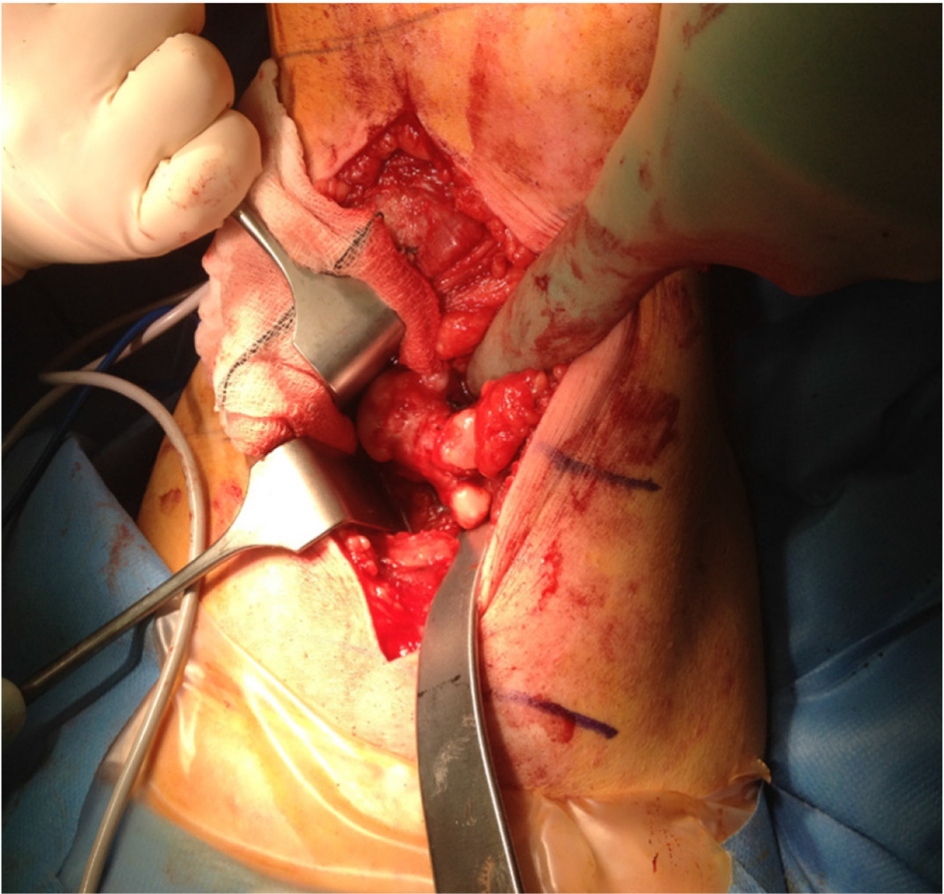
A popliteal exploration was undertaken and preservation of structures such as the popliteal artery and vein, the common peroneal nerve and the tibial nerve was performed.

The patient underwent a 3.5-h operation by a team of vascular and orthopaedic surgeons who initially performed a popliteal exploration and preservation of vessels such as the popliteal artery and vein, and the common peroneal nerve and tibial nerve (Figure 2).

**Figure 3.**
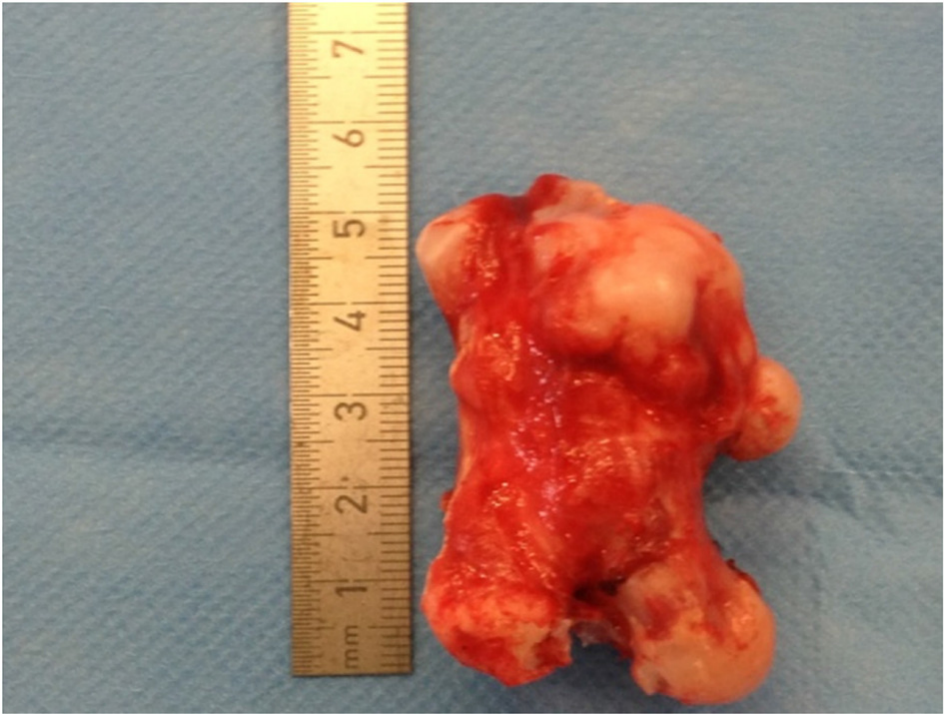
Histological assessment confirmed a benign irregular shaped fungating bony lesion 55 × 25 × 44 mm in keeping with an osteochondroma.

**Figure 4.**
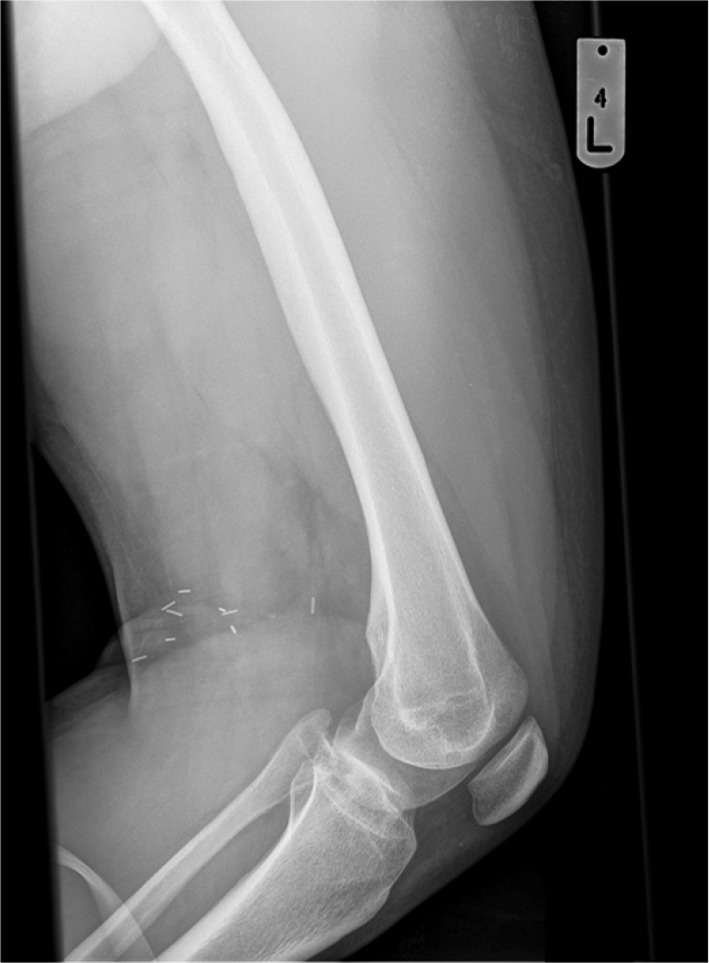
Follow-up imaging 5 years post-operatively ruled out any reoccurrence of bony growth.

The osteochondroma was then identified and excised. Histological findings confirmed the lesion to be an osteochondroma (Figure 3). No malignant transformation was reported. A 1 year follow up of the patient in the orthopaedic outpatient service showed resolution of the clinical features of deep vein thrombosis. A 5 year follow up X-ray image showed no re-growth at the site of excision (Figure 4).

## Discussion

DVT in a young, otherwise healthy mobile person is extremely uncommon. Among 20–40 year olds in Western population, as few as 1/100,000 will develop a DVT.^1^ This is 10 times lower than the overall population incidence.^2^ DVT has both acquired and genetic aetiologies.^3^ In this case hypercoagulability phenomena and genetic disorders were ruled out. We therefore had to consider acquired causes that may lead to venous stasis and cause recurrent DVTs in a fit young man.

**Figure 5.**
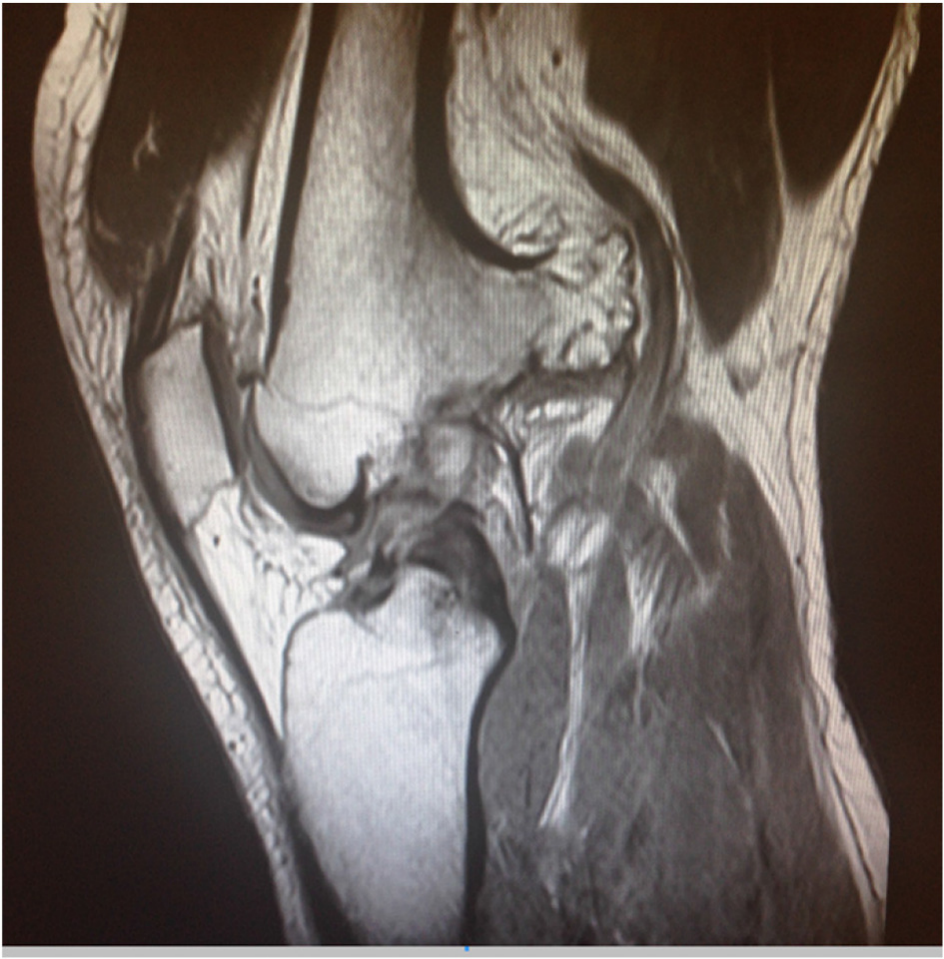
Sagittal MRI imaging of the left knee demonstrates the extent of the osteochondroma and the impact on surrounding structures.

Osteochondroma, or exostosis, is the most frequent benign tumour of bone occurring in 1% of the population. An osteochondroma is a cartilage-capped benign bony tumour arising from the external surface of bones. They are commonly found in the metaphyseal regions of long bones and protrude away from the epiphysis. They are often long and slender, and pedunculated on a stalk that is continuous with the underlying cortex and cancellous bone and are protected by a cartilaginous cap.^4^ Due to their irregularity, optimal imaging for characterization and evaluation is by MRI (Figure 5).^5^ MRI can also calculate cartilage cap thickness and as a result, offer insight into the risk of malignant transformation. A high level of suspicion of malignant transformation should be maintained when the cartilage thickness of an osteochondroma is greater than 15 mm in a skeletally mature patient.^4^ MRI can also delineate surrounding structures, vessels and soft tissue and determine the impact of the osteochondroma on these.

The majority of osteochondromas are asymptomatic and diagnosis is usually incidental or related to complications in 4% of cases such as bursa formation, malignant transformation, neurogenic compromise or vascular complications.^5^ Osteochondromas can occur as solitary or multiple tumours. When they present as multiple tumours they are often a consequence of an autosomal dominant predisposition—in this setting, numerous osteochondromas can result in skeletal deformity.^6^ When multiple, the risk of malignant transformation increases 10-fold.

Vascular complications are rare and are predominantly arterial,^7^ occurring when there is an abnormally short distance between the exostosis and the advential vessel wall which can lead to friction and tearing. Resulting complications such as the development of a non-pulsatile (24.4%) or pulsatile mass (38.6%) due to an underlying pseudoaneurysm, distal ischaemia (24.4%) and phlebitis (2.2%) have been reported.^6^ These conditions are more commonly found in male adolescents and young adults, corresponding to the age at which growth ceases and ossification of the cartilaginous spike occurs, and may be related to strenuous physical exercise.^8^

As this case demonstrates, vascular complications are not limited to the arterial network. Pseudoaneurysm from an adjacent artery and mass effect of a large volume exostosis can lead to venous occlusion and ultimately deep venous thrombosis.^9–12^ An isolated DVT with no arterial involvement, however, is far less common. To our knowledge, there are fewer than five cases identifiable in the literature. In this case, a multidisciplinary approach to diagnosis and management of the patient was crucial. Radiological reports indicating that there was bowing of vessels over the exostosis signalled the complicated surgery required to safely resect the bony tumour while maintaining the integrity of surrounding structures.

Vasseur et al highlighted that vascular interruption occurs secondary to a osteochondroma should be managed early with operative excision to prevent vascular complications including distal vessel disease if arterial, or phlebitis and pulmonary emboli if venous.^6^ Surgery, in the absence of symptoms, is not recommended, unless there is growth disturbances or suggestion of the lesion harbouring malignant transformation potential, as is often the case with multiple hereditary osteochondromas.

## Conclusions

To date, there are very few cases of an isolated DVT secondary to osteochondroma documented in literature. Osteochondroma is usually an incidental finding on a radiograph that rarely requires intervention. This case reiterates the value of imaging in the diagnosis and management of a surgical rarity.

## Learning points

1. This case report provides an overview of how DVT secondary to osteochondroma formation presents and how it is appropriately diagnosed and managed.
2. Appropriate imaging throughout the management of a surgical rarity can provide guidance for a structured management pathway, pre –, peri – and post-operatively.
3. The importance of a multidisciplinary approach is crucial. The vast range of possible causes of deep vein thrombosis encouraged a multifocal approach which assisted in identifying the osteochondroma as the predisposing agent to the venous occlusion.
4. Timely and appropriate imaging can be the key to diagnosis.
5. Follow up interval radiographs can help rule out recurrence of osteochondromas.

## Consent

Written informed consent for the case to be published (including images, case history and data) was obtained from the patient(s) for publication of this case report, including accompanying images.
